# Maturation, Refinement, and Serotonergic Modulation of Cerebellar Cortical Circuits in Normal Development and in Murine Models of Autism

**DOI:** 10.1155/2017/6595740

**Published:** 2017-08-15

**Authors:** Eriola Hoxha, Pellegrino Lippiello, Bibiana Scelfo, Filippo Tempia, Mirella Ghirardi, Maria Concetta Miniaci

**Affiliations:** ^1^Neuroscience Institute Cavalieri Ottolenghi (NICO), Torino, Italy; ^2^Department of Neuroscience, University of Torino, Torino, Italy; ^3^Department of Pharmacy, University of Naples Federico II, Naples, Italy; ^4^National Institute of Neuroscience (INN), Torino, Italy

## Abstract

The formation of the complex cerebellar cortical circuits follows different phases, with initial synaptogenesis and subsequent processes of refinement guided by a variety of mechanisms. The regularity of the cellular and synaptic organization of the cerebellar cortex allowed detailed studies of the structural plasticity mechanisms underlying the formation of new synapses and retraction of redundant ones. For the attainment of the monoinnervation of the Purkinje cell by a single climbing fiber, several signals are involved, including electrical activity, contact signals, homosynaptic and heterosynaptic interaction, calcium transients, postsynaptic receptors, and transduction pathways. An important role in this developmental program is played by serotonergic projections that, acting on temporally and spatially regulated postsynaptic receptors, induce and modulate the phases of synaptic formation and maturation. In the adult cerebellar cortex, many developmental mechanisms persist but play different roles, such as supporting synaptic plasticity during learning and formation of cerebellar memory traces. A dysfunction at any stage of this process can lead to disorders of cerebellar origin, which include autism spectrum disorders but are not limited to motor deficits. Recent evidence in animal models links impairment of Purkinje cell function with autism-like symptoms including sociability deficits, stereotyped movements, and interspecific communication by vocalization.

## 1. Introduction

The neuronal architecture of the mature nervous system is reached through complex processes of synaptic rearrangement during pre- and early postnatal life. This process is established by a genetic program and is then refined by experience, activity, and molecular cues. During this critical period, the developing brain displays an extraordinary degree of plasticity and undergoes an extensive refinement of the neuronal network consisting in the removal of redundant inputs (synapse elimination) and strengthening of the remaining ones (for review see [1–3]). The cerebellum represents an attractive model to study the cellular and molecular mechanisms that underlie neural remodeling in the developing brain. Even subtle defects in this process are responsible for disorders due to improper signaling in the cerebellar neuronal network, ranging from symptoms belonging to the autism spectrum to classical ataxic motor deficits. A high degree of plasticity is retained in mature cerebellar circuits. Such adult synaptic rearrangements are essential for a continuous updating and refinement of data processing, which are the basis of cerebellar learning and memory.

## 2. Structural Plasticity in the Developing Cerebellum

In the adult cerebellar cortex, parallel fibers (PF), originating from granule cells, establish more than 100,000 synapses on the distal dendritic compartment of Purkinje cells (PC) while each PC is innervated on the proximal dendritic domain by a single climbing fiber (CF), which is an axonal branch of an inferior olivary neuron [4, 5]. This exclusive projection is achieved during development by drastically reducing the number and reshaping the distribution of the olivocerebellar CFs. At birth, each PC receives innervation by multiple CFs with similar synaptic strength [6–8]. These redundant CFs are eliminated during the second and third postnatal weeks, attaining monoinnervation by postnatal day 20 (P20) in mice (for review, see [9–11]). The single predominant CF forms hundreds of excitatory synapses, whose activation evokes, in PC dendrites, a strong depolarization and a pronounced increase in intradendritic calcium concentration, mediated by voltage-dependent Ca^2+^ channels (VDCCs) of the P/Q type [12–16].

The development of CFs has been classified in several stages [17, 18], starting from the “creeper” stage (P0), in which CFs crawl among PC somata to form transient synapses on immature dendrites, followed by the “pericellular nest” stage (P5) during which CFs display a high synaptogenic activity on PC somata. Then, CF innervation is displaced to the apical portion of PC somata in the “capuchon” stage (P9) and translocates to the dendrites in the “dendritic” (P12) stage. In parallel with the removal of CF synaptic terminals on the PC soma, GABAergic synapses from basket cells and stellate cells are massively formed on PCs [19–21].

As the PC dendritic arbor develops, the CF-PC connection is refined with the extension of a few CFs and the retraction of the others with the final outcome of a single winner CF and the complete withdrawal and disappearance of all the others. This process consists of at least two distinct phases, an earlier phase up to P7 where multiple CFs establish their synapses onto the PC and a later phase from P7 to P21, in which all CFs except one are eliminated [22–25]. The strength of different CFs in a single PC starts to diverge already in the early phase, indicating that an ongoing competition between CFs already started at this time [23]. While the early phase is independent of PF-PC synapse [11, 26], the later phase requires normal PF-PC synaptic transmission [11, 26] and involves heterosynaptic and homosynaptic mechanisms of CF elimination [7, 8, 22, 26–29]. The early phase of CF development is also influenced by the patterns of olivary activity, as shown by persistence of multiple CFs in PCs until at least 3 months of age following administration of harmaline, which abnormally synchronizes olivary neuron activity [30]. Postsynaptic activity also plays a role in this process: reducing PC excitability by expression of a recombinant chloride channel during the second postnatal week prolongs CF multiple innervation until adulthood [31].

A critical role of the P/Q-type VDCCs has been reported in CF homosynaptic competition leading to selective strengthening of single CF inputs, promotion of dendritic translocation of the strengthened CFs, and subsequent CF synapse elimination [15, 32, 33]. Mice with a selective deletion of Cav2.1 in PCs show an impaired heterosynaptic competition between PFs and CFs on PCs, which leads to hyperspiny transformation and chaotic innervation by multiple CFs and numerous PFs in proximal somatodendritic domains of PCs, and progressive degeneration of PCs [16]. A similar disruption of synaptic wiring is also induced by chronic blockade of neuronal activity by tetrodotoxin (TTX) and of AMPA receptors by NBQX in mature cerebellum [34–36]. In addition, Kakizawa et al. [37] reported that CF synapse elimination from P8 to P12 is modulated by local application of insulin-like growth factor I, suggesting that IGF-1 may provide a trophic support to maintain CF synapses. While the early phase of CF elimination is mainly driven by homosynaptic competition, in the late-phase of CF elimination, heterosynaptic regulations by PFs and GABAergic synapses have a crucial role.

The formation and maintenance of the PF-PC synapse is modulated by the GluD2 receptor (previously called GluR*δ*2 [38, 39]) that is selectively expressed at this synapse but not at the CF synapse [40, 41] and has a specific site of interaction (the N-terminal domain) with the presynaptic neurexin via the synaptic organizer Cbln1 [42–44]. GluD2 belongs to ionotropic glutamate receptors but it is not gated by glutamate. The gate of GluD2 has been shown to be opened by activation of the type 1 metabotropic glutamate (mGlu_1_) receptor [45, 46]. D-Serine released from astrocytes also binds to GluD2, with an inhibitory effect on LTD induction [47]. During development, GluD2 is involved in the stabilization and strengthening of the synaptic connection between PFs and PCs [48–50].

Mutant mice deficient in the GluD2 receptor [28, 29] exhibit impaired formation and stabilization of PF-PC synapses and abnormalities in CF innervation patterns with multiple CF innervation not only around the PC soma but also in PC distal dendrites where PFs normally form synapses. The aberrant dendritic innervation was also observed in the mice with hypogranular cerebellum generated by methylazoxy methanol acetate treatment [51] and in mutant mice deficient in Cbln1 [42] that binds to GluD2 and stabilizes PF-PC synapses.

The impairment of the late phase of CF synapse elimination has also been observed in mice deficient in the mGlu_1_ receptor-G*α*q-PLC*β*4-PKC*γ* signaling cascade in PCs [52–57] and after chronic blockade of cerebellar NMDA receptors [58, 59].

GABAergic inhibition is also crucial for CF synapse elimination as reported by Nakayama et al. [60] in mice with a heterozygous deletion of the GABA-synthesizing enzyme GAD67 that results in a reduced activation of GABA_A_ receptors within the cerebellum. The association of CF synapse elimination and alteration in GABAergic transmission has also been revealed in TrkB-deficient mice [61–65].

**Figure 1:**
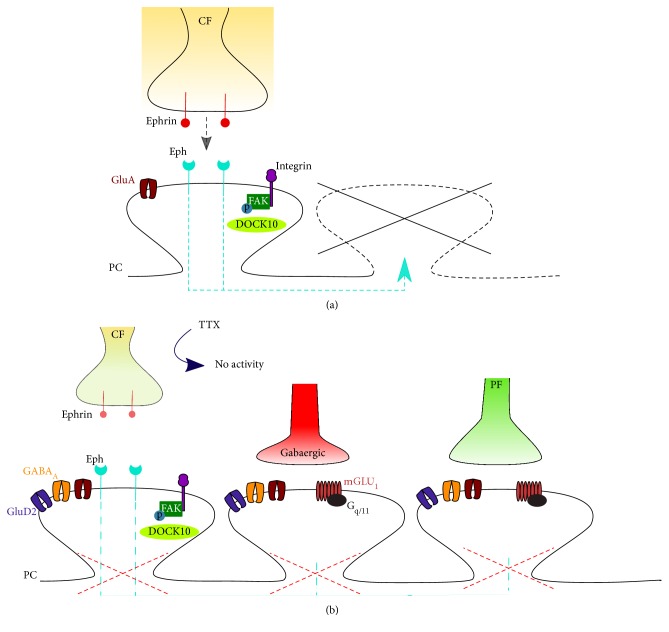
(a) Schematic model of the mechanisms of the ephrin pathway in the control of CF-induced spine regulation in mature cerebellar PCs (see text). (b) The block of activity induces proliferation of spines expressing GluD2 and GABA receptors, atrophy and loss of contacts of the CF, and expansion of the PFs and GABAergic terminals to the proximal domain of PCs.

Elimination of redundant climbing fibers to PC synapses in the developing cerebellum is also regulated by retrograde signals from postsynaptic cells, such as semaphorin3A and semaphorin7A [66, 67]. Indeed, the knockdown of Sema3A in PCs or its coreceptor, plexinA4, in CFs accelerated CF synapse elimination and decreased CF-mediated synaptic inputs. Conversely, knockdown in PCs of Sema7A or in CFs of either of the two receptors for Sema7A, plexinC1, and integrinB1 impaired CF synapse elimination (Figure 1). Importantly, the effects of Sema7A involve the activation of mGlu_1_ signaling, which is likely involved in the late stage of CF synapse elimination.

Therefore, in the first 3 weeks of postnatal development in rodents, homosynaptic competition among CFs, heterosynaptic competition between CFs and PFs, and retrograde signals from the PC promote the maturation of PC circuitry in the cerebellar cortex, with strict territorial innervation by CFs and PFs and monoinnervation of PCs by CFs.

## 3. Structural Remodeling in the Mature Cerebellar Cortex

The structural plasticity that allows the formation of the highly organized excitatory wiring in early postnatal development is remarkably well maintained in the mature cerebellar cortex. In the mature cortex, PCs also receive a strong inhibitory GABAergic input from basket and stellate neurons, which are distributed along the PC's somatodendritic region.

The proximal dendritic compartment of PCs has a very low number of spines, which are organized in clusters and postsynaptic to CF varicosities, while the distal compartment is contacted by thousands of PFs and has a very high spine density (spiny branchlets) [4]. In the mature PC, GluD2 receptors are selectively localized in dendritic spines postsynaptic to PFs [41, 68].

Structural changes of PC dendritic spines represent a fundamental aspect of the mature cerebellar cortex plasticity. Dendritic spines are considered the major loci of synaptic plasticity in the brain and therefore the structural substrate of memory [69–74]. Several studies showed that dendritic spines are highly dynamic structures, which can change their shape and size. In response to electrical and chemical stimuli, dendritic spines can be retracted or can be generated anew. This extraordinary plasticity is present throughout life, suggesting that the dynamic assembly of spines is essential for normal brain function [75–82]. In different brain regions, the rate of spine turnover is modulated by sensory experiences and learning [83–85] and altered in some pathological states including autism and depression [86–90]. Several evidences show that spine growth can be induced by presynaptic terminals or they can form by intrinsic mechanisms, as occuring in PCs [69, 91, 92].

The two main excitatory inputs, CF and PF fibers, that contact the proximal and distal domains, respectively, of the PC dendrites compete to keep their innervation territory during development and in adulthood. This competition appears to be regulated by activity and determines and maintains the adult phenotype of PCs. Studies in adult rats have demonstrated that a few days after a subtotal lesion of the inferior olivary neurons induced by 3-acetylpyridine, the surviving CFs are able to sprout and reinnervate the denervated PCs [93, 94] while a large number of new spines emerge from the PC's proximal dendritic domain accompanied by sprouting of nearby located PFs [95, 96]. This indicates that, as one of the two inputs is weakened, the other one takes over the territory left. Interestingly, GluD2 receptors appear in the newly formed spines innervated by PFs and CFs [97]. Reactive PC hyperspinogenesis and synaptogenesis by the PF input have also been reported following lesion of olivocerebellar axons [98].

After PC degeneration induced by kainic acid or propidium iodide or by genetic mutations, CFs progressively undergo remarkable regressive modifications with the disappearance of most of their terminal arborisation [99, 100]. However, these structural and functional changes are reversed whenever embryonic PCs are transplanted into a kainic acid lesioned or into a mutant cerebellar cortex [96, 101–104].

Blocking electrical activity in adult cerebellar cortex by infusing TTX or a selective AMPA receptor antagonist induces the loss of a large number of synaptic contacts of CF terminal arbors, the expansion of the PF territory which invade the proximal dendritic domain of PCs, and the appearance of a high number of new spines in PC proximal dendrites [34, 36, 97, 98] that express GluD2 receptors. Interestingly, this receptor is also expressed in spines innervated by GABAergic neurons and in those still in contact with the CF, suggesting that GluD2 expression is an intrinsic activity-independent property of all PC spines that is independent from the type of afference [105]. The changes were reversible upon removal of the TTX block [34, 105]. These findings led to the hypothesis that the CF exerts an activity-dependent spine-pruning action with downregulation of GluD2 expression in its own spines at the proximal dendrites around its synapses as a kind of lateral inhibition [97, 106] through ionotropic AMPA/kainate receptors [36].

After the block of electrical activity, the density of GABAergic terminals is significantly increased only in the PC proximal domain [107] with the appearance of asymmetric spine synapses expressing GABA_A_ receptor subunits in addition to glutamate receptors and GluD2 subunits. These observations suggest that the competition for the innervation of PCs is not limited to the two excitatory inputs but the activity of the CF also has a fundamental role in the maintenance of the proper synaptic excitatory and inhibitory architectural wiring.

The repression of spine proliferation induced by CF activity in PC proximal dendrites may be mediated by Eph receptor signaling [108], a pathway that plays an important role in dendritic spine formation and maintenance [109–112]. Cesa et al. [108] showed that the inhibition of both ephrin A and ephrin B induced a rapid proliferation of spines in the proximal dendrite, while infusion of ephrin A2 or ephrin B1 partially suppressed the proliferation of proximal spines that occurs when CF electrical activity is blocked *in vivo* by TTX. Interestingly, animals lacking ephB1, B2, and B3 exhibited a significantly high spine density in the PC proximal dendrite despite the presence of CFs, suggesting that one or more of these receptors is the target on PCs for ephrins released from the CFs.

Jaudon et al. [113] suggested that among the possible downstream targets of Eph receptors in the cerebellum, there are the members of the DOCK family of RhoGEFs such as the Cdc42-specific GEF DOCK10. They found that DOCK10 protein expression increased during PC development whereas depletion of DOCK10 in PCs leads to dendritic spine defects. In addition, they reported that DOCK10 is essential for spine formation, not only in PCs but also in cultured hippocampal neurons. Interestingly, a human genetics study showed that deletion of the *DOCK10* gene is associated with autism spectrum disorders [114] characterized by developmental alterations of spines and loss of synaptic plasticity.

Recently Heintz et al. [115] suggested that Eph/ephrin signaling regulates proximal dendritic spines in PCs by inactivating integrin downstream signaling, that is, known to be involved in spine formation, stability, motility, and morphology [82, 116–119]. In mixed cerebellar cultures deprived of CFs, they demonstrated that Ephrin A3 inhibits integrin activity in proximal dendritic spines and induces a collapse of proximal but not distal spines, similar to the effect of CFs, which is prevented by integrin activation.

These findings suggest an interesting mechanism action by which CFs can shape the proximal dendrites of PCs and remodel the spine distribution, from the numerous small spines typical of distal dendrites to the few large spines that contact CFs. This process is likely regulated by ephrin, which is released either by the CF or by perisynaptic astrocytes. Ephrin acts on EphA4 on PC dendrites, leading to a signaling process, restricted to the proximal dendrites, that inactivates integrins or focal adhesion kinase (FAK) in the spines, causing spine retraction (Figure 1) [115].

## 4. The Role of the Serotonergic System in the Development, Maturation, and Refinement of Cerebellar Synaptic Networks

Considerable evidence supports the idea that serotonin (5-HT) acts as a regulator of brain development and contributes to the refinement of neuronal circuitry during the critical periods of early postnatal life [120–124]. Alterations of the 5-HT system during early development are considered to play a critical role in the etiology of neurodevelopmental disorders such as autism and schizophrenia [125, 126].

Serotonergic projections to the cerebellum develop during the postnatal period in coincidence with the perinatal development of the cerebellar cortex [127]. Developmental events such as PC maturation, climbing fiber elimination, and granule cell migration are orchestrated processes that are likely modulated by the 5-HT system.

The variety of 5-HT effects in developmental processes is mediated by different types of 5-HT receptors (5-HTR), each with its spatial and temporal expression pattern [128, 129]. Thus by far, 14 subtypes of 5-HTRs have been identified and classified into seven families and designed 5-HT_1_R through 5-HT_7_R, based upon their structure and pharmacological profile. Six of the families (5-HT_1,2,4–7_) are members of the G-protein-coupled receptor superfamily, whereas 5-HT_3_ belongs to the superfamily of ligand-gated ion channels [130].

**Figure 2:**
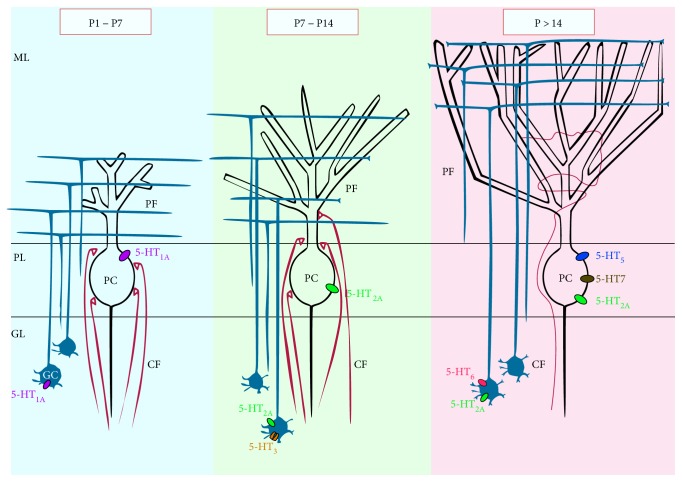
Schematic representation of the 5-HT receptor subtypes expressed by PCs and granule cells (GCs) during the different stages of postnatal cerebellar development. GL: granular layer; PL: Purkinje cell layer; ML: molecular layer.

The expression of 5-HTR subtypes and therefore the effects of 5-HT in the developing cerebellum vary with the stage of cell differentiation [131–133] (Figure 2). In particular, 5-HT_1A_Rs are transiently expressed by cerebellar granule cells during the first 2 weeks and no expression is detected in adult rodents [134]. On the other hand, 5-HT_2A_Rs are expressed on granule cells late in development: expression of 5-HT_2A_Rs starts from P5, increases dramatically at the second postnatal week, and remains sustained, at a lower level, until 10 weeks of age. In PC, the time course of expression of 5-HT_1A_ and 5-HT_2_ receptors is almost similar to that of granule cells. The 5-HT_1A_R is detected on PCs only in the first postnatal week. 5-HT_2A_R appears in the PC soma shortly after birth and increases first in the proximal dendrites of PCs at P6–P10 and then in proximal and distal dendrites after P12, where it is maintained in adulthood [135]. 5-HT_1A_ and 5-HT_2A_ receptors have different effects on the development of PC dendrites. Studies *in vitro* have demonstrated that 5-HT promotes dendritic growth of PCs through 5-HT_1A_Rs, while 5-HT_2A_Rs inhibit the dendritic development of PC [136]. These data are in accordance with other *in vivo* and *in vitro* studies showing an important role of 5-HT in dendritic development, formation of dendritic spines, and synapse formation in the cerebral cortex and hippocampus [137].

Expression of 5-HT_3_ receptors has been observed in granule cells of the cerebellum within the first three postnatal weeks in rodents with relatively high expression in lobules I–VI and to a lesser extent in lobules VII–X [138, 139]. Studies in 5-HT_3A_/EGFP transgenic mice have revealed that the expression pattern of 5-HT_3_R at P7 follows the migration pathway of the cerebellar granule cells from the external to the internal granule cell layer; then, by P14, the expression starts to decrease and becomes completely absent after P21 [138]. In contrast, no coexpression of 5-HT_3_ receptors has been found with glial cells, PCs, and interneurons. Patch clamp recordings from granule cells of 5-HT_3A_R/EGFP transgenic mice indicated that 5-HT_3_ receptors are in a functional state at P6-P10, since application of a selective 5-HT_3_ receptor agonist induced a fast inward current. At the same age, functional 5-HT_3_ receptors appear also at the presynaptic site of the PF-PC synapse, where they modulate short-term synaptic plasticity and eventually the maturation of these synapses. Interestingly, the time window of 5-HT_3_ receptor expression corresponds to the period during which PC dendrites develop [140]. The involvement of 5-HT_3_ receptors in controlling the morphological maturation of PCs has been clearly demonstrated by Oostland et al. [139] in slices of 5-HT_3A_R knockout mice at P9 and in organotypic slice cultures treated at P8 with a 5-HT_3_R antagonist. In both models, PCs show a higher dendritic length and complexity with respect to the control condition. In addition, 5-HT_3_R knockout animals show delayed CF elimination from P7 to P24, whereas no difference in the number of climbing fibers innervating one Purkinje cell was observed at P5 and P6 compared to controls. Morphology and physiology of PCs in 5-HT_3_R knockout mice appear normal in adult mice. This suggests that 5-HT_3_Rs regulate the maturation of the cerebellar circuitry during a specific time window when heterosynaptic competition between parallel fibers and climbing fibers occurs.

Based on the different temporal expression pattern of these 5-HTR subtypes, it has been argued that, during the first postnatal week, activation of 5-HT_1_Rs expressed by both granule cells and PCs promotes PC dendritic growth [139]. Later activation of 5-HT_3_Rs expressed by granule cells limits dendritic growth of PCs and controls the CF elimination from PC dendrites. Last, 5-HT_2_Rs expressed by granule cells and PCs determine inhibition of PC dendritic growth and promote synaptic activity stability.

Anatomical and pharmacological studies indicate that the cerebellar cortex expresses additional subtypes of 5-HTRs in both the developing and adult cerebellum. For example, 5-HT_5_ and 5-HT_7_ subtypes have also been found on PCs, whereas 5-HT_6_Rs are expressed on granule cells [141–144]. Expression of 5-HT_5A_R has also been found on Golgi cells and molecular layer interneurons [134, 142].

The cerebellar widespread projection of 5-HT fibers and the variety of 5-HT receptors allow the 5-HT system to differentially modulate both excitatory and inhibitory synaptic transmission throughout the entire cerebellar cortex until adulthood [145, 146]. Indeed, studies *in vitro* have demonstrated that application of 5-HT facilitates the GABAergic transmission between cerebellar interneurons (i.e., basket, stellate, and Lugaro cells) and PCs [147, 148] but reduces the release of the excitatory transmitter glutamate from PFs onto PCs [149]. This suggests that 5-HT determines an overall depression of PC activity via suppression of excitatory inputs from PFs and facilitation of inhibitory inputs from interneurons. Such mechanism can potentially decrease the inhibitory drive of PCs to deep cerebellar nuclei neurons and ultimately refine the motor output.

Lippiello et al. [144] have recently demonstrated that in adult mice, 5-HT also exerts a fine regulation of synaptic plasticity at the PF-PC synapse via 5-HT_7_Rs, without affecting the CF-PC synaptic transmission. Indeed, the selective activation of 5-HT_7_R by LP-211 caused, on one hand, LTD of the PF-PC synapse and, on the other hand, impaired LTP at the same synapse. Moreover, bath application of a 5-HT_7_R antagonist prevented LTD produced by pairing PF stimulation with PC depolarization. The suppressive effect of LP-211 on LTP induction can be considered an important mechanism to prevent the simultaneous occurrence of conflicting forms of plasticity, such as potentiation of synaptic transmission under conditions that promote postsynaptic LTD. The combination of LTP and LTD is believed to underlie several forms of motor learning [150]. The involvement of serotonin in motor learning has been observed in several cerebellar-dependent paradigms. For example, depletion of brain 5-HT has been shown to impair adaptation of the horizontal vestibulo-ocular reflex (VOR) in rabbits [151]. On the other hand, activation of 5-HTR improves motor coordination deficits in patients with inherited or acquired ataxia.

## 5. Cerebellar Network Maturation and Refinement in Autism Spectrum Disorders

Autism spectrum disorders (ASD) are characterized by deficits in social interaction, impaired communication, repetitive behaviors, and restricted interests [152]. In the last decades, it has been recognized that most cases of ASD are associated with cerebellar malformations (for review, see [153]). The loss of PCs is the single most frequent finding at autopsy of subjects with ASD [154, 155]. Recent studies in animal models of ASD have clearly shown that the cerebellar cortex is a pivotal structure in the pathogenesis of autism. The experimental strategies of these studies include selective gene deletion in cerebellar PCs [156–158] and reversal of autistic symptoms by re-expression, in PCs, of a wild-type gene [159].

The PC-selective deletion of the *Tsc1* gene (*Tsc1^PC^*) in mice reproduces the core symptoms of autism that in this animal species are displayed as impairment of sociability, repetitive grooming behavior, and deficit of vocalization [156]. While homozygous *Tsc1^PC^* mice also display PC loss and motor symptoms, heterozygous ones display ASD symptoms without other accompanying signs. In such heterozygous *Tsc1^PC^* mice, PCs have a normal overall morphology but show increased PC spine density, which is in line with the frequent finding of hyperspiny neurons in ASD patients [160]. This feature has been attributed to a failure to complete the maturation of dendritic spines [161]. In *Tsc1^PC^* mice, PCs show a reduced spontaneous and evoked action potential firing, although synaptic transmission is unaltered. Although the reason of the impairment of PC firing is unknown, it might affect, via deep cerebellar nuclei, downstream cerebral networks thereby causing autistic symptoms.

A second, independent murine model of ASD was generated by selective deletion of *phosphatase and tensin homolog missing on chromosome 10* (*Pten*) in cerebellar PCs (*Pten^PC^* mice) [157]. *PTEN* mutations are responsible for up to 5–10% of ASD cases [162]. PTEN has an antagonistic action on phosphatidylinositol-3-kinase (PI3K), with a negative regulation of its transduction pathways. Interestingly, the PI3K/PTEN signaling pathway regulates the TSC1/TSC2 complex and its downstream targets, including the mammalian target of rapamycin (mTOR) [163, 164]. *Pten^PC^* mice show PC degeneration starting at 6 months of age, but autistic-like symptoms are present before the onset of PC death. They display impaired sociability and repetitive behavior (jumping/scrabbling). PCs of *Pten^PC^* mice show several structural and functional abnormalities. PC soma, dendrites, and axon are enlarged, with focal dendritic swellings and axonal torpedoes. PCs of *Pten^PC^* mice showed a lower spontaneous firing rate and reduced excitability, similar to *Tsc1^PC^* mice. These deficits were associated with a lower input resistance, which might be at least partially responsible for the reduced excitability. However, in contrast to *Tsc1^PC^*, in which PC postsynaptic currents were intact, in *Pten^PC^* mice, the excitatory postsynaptic currents (EPSCs) evoked by PF activity (PF-EPSCs) were aberrantly larger and those evoked by CF activity (CF-EPSCs) were smaller than in controls. These functional alterations might co-operate to determine improper output signals responsible for the development of autism.

A third model of ASD is the *Shank2*-knockout mouse [158, 165]. Mutations of *SHANK2* are associated with ASD, and *SHANK2* variants in conserved amino acids are enriched in individuals with ASD [166]. Previous studies had shown that a global deletion of *Shank2* causes ASD-like behaviors [167, 168]. Shank2 is localized at the postsynaptic density and is involved in synaptic formation and refinement [165]. A PC-selective deletion of *Shank2* (*Shank2^PC^*) has been generated by two research groups, with different results. Peter et al. [158] found that *Shank2^PC^* mice show deficits in social interaction. In contrast, Ha et al. [165] report normal sociability and vocalization and absence of repetitive behavior in their *Shank2^PC^* mice. In global *Shank2* knockout mice, the density and morphology of PC dendritic spines were intact [158, 165]. In contrast, in the same mice, Ha et al. [165] found a significant reduction in the number of postsynaptic densities. Both studies report a reduced expression of glutamate receptors in dendrites of global *Shank2* knockout PCs, suggesting a deficit in formation and maintenance of glutamatergic synaptic contacts. On the other hand, in *Shank2^PC^* mice, only GluD2 (GluRD2) and PSD93 were significantly reduced [165]. Evoked PF-EPSCs had normal amplitude [158]. However, Ha et al. [165] analyzed spontaneous EPSCs in *Shank2^PC^* PCs and found a markedly lower frequency, in line with a reduced number of excitatory synaptic contacts. Peter et al. [158] also analyzed, but in global *Shank2* knockout PCs, spontaneous GABAergic IPSCs, which were increased in frequency. The PC spontaneous firing recorded *in vivo* from *Shank2^PC^* mice had a normal frequency but a higher variability [158]. The synaptic plasticity of the PF-PC synapse was studied by Peter et al. [158] in the global knockout and by Ha et al. [165] in *Shank2^PC^* PCs. In both models, long-term depression (LTD) was preserved, while long-term potentiation (LTP) was only analyzed in the global knockout, where it was impaired [158]. It remains to be determined whether LTP is impaired also in the PC-selective knockout model. Taken together, the results on *Shank2^PC^* mice indicate a variable influence on autistic-like behaviors and only subtle synaptic and firing alterations. An overview of the result on these PC-selective models is reported in Table 1.

The critical periods in which the causative or risk genes produce neural functional alterations responsible for autism are not known. Studies of patients with ASD provided compelling evidence that the onset of the disease is in the first months of life, even if the diagnosis is often delayed [169]. However, gene expression studies showed that the peak of expression of the majority of risk genes converges during fetal development, while for another subset of genes, it coincides with neuronal maturation [170]. This problem has been studied in animal models by conditional gene expression utilizing inducible mutations [171]. Since ASDs are a heterogeneous class of disorders, in line of principle, it is possible that some cases are reversible even in the adult stage, while others are expected to be refractory to any kind of treatment outside a critical period in which the genetic defect triggered the neural alterations. The former case has been shown following deletion of *Mecp2* in adult mice, which impairs the nest-building behavior, as in germinal knockouts [172]. Reactivation of *Mecp2* in adulthood rescues this behavioral deficit [173]. However, it must be pointed out that in this model, no other ASD-linked behavior is altered. In contrast, in a murine model of the Angelman syndrome, which includes ASD symptoms, a rescue of *Ube3a* expression is only effective in embryonic age, while its re-expression during postnatal life fails to recover the autism-related behaviors [174]. In animal models of ASD caused by mutations of the synaptic gene *Nrxn1β* or *Shank3*, the re-expression of the defective transcript in adulthood successfully reversed the ASD-related behaviors, including impaired sociability and increased grooming [175, 176].

The developmental time windows, in which cerebellar functional alterations cause ASDs, are not known. It is interesting to note that a speech-language disorder caused by mutations of *FOXP2* is associated with impairment of PC dendritic development. A murine model of this disease, in infantile age, presents reduced ultrasonic vocalizations [177, 178], an oral form of communication often compromised in ASD models. The conditional re-expression of *Foxp2* in the cerebellum is sufficient to rescue this deficit [159]. Thus, also in this form of interindividual communication, the cerebellum plays a pivotal role, which in this case is critical in neonatal age. Future studies are necessary to establish the critical periods for the cerebellar induction of the various forms of ASD.

## 6. Conclusions

In spite of the regularity of the cellular and synaptic organization of the cerebellar cortex, the mechanisms by which the mature wiring is attained are very complex. For the CF-PC synapse alone, multiple intrinsic and extrinsic signals are involved and interact with specific temporal and spatial patterns. The serotonergic system plays a crucial role in enabling and in fine tuning this process of synaptic organization. Recently, it has been shown that genetic modifications selectively targeted to PCs are sufficient to generate autism-like symptoms in animal models, by interfering with these delicate and complex developmental programs. In line with the important role of 5-HT in orchestrating the synaptic organization of the cerebellar cortex, the serotonergic system is also involved in autism. Indeed, 30–50% of autistic subjects show elevated blood levels of serotonin [179], which interfere with the developmental process of brain areas including cerebellum. Indeed, it has been demonstrated that pre- and postnatal exposure to abnormally high levels of the serotonergic agonist, mimicking the levels observed in autism, induced a significant decrease in the total number of dendritic spines in neurons of the dentate nucleus of rat cerebellum [180]. In addition, analysis of postmortem tissues has revealed a significant impairment in the activity of monoamine oxidases-A (MAO-A), in the cerebellum of children with autism (age 4–12 years) compared to control subjects [181]. Since monoamine oxidases (MAOs) catalyze the metabolism of monoamine neurotransmitters, a reduced MAO-A activity will cause an increase of 5-HT level in the brain of autistic subjects. Interestingly, MAOA knockout mice exhibit increases in brain serotonin levels, as well as abnormally high fear conditioning, enhanced eye-blink conditioning, and increased LTP in the hippocampus [182, 183].

Future studies are necessary to better understand the mechanisms of synapse formation refinement of the cerebellar network and how inputs are compartmentalized to enable proper functioning and avoiding disorders ranging from motor control deficits to autism. Furthermore, studying the complex effects of 5-HT on synaptogenesis and establishment of cerebellar cortical networks, and the corresponding behavioral and psychiatric phenotypes, may provide new therapies for early intervention on neurodevelopmental disorders.

## Figures and Tables

**Table 1:** 

	*Tsc1^PC^*	*Pten^PC^*	*Shank2^PC^* Peter et al. [158]	*Shank2^PC^* Ha et al. [165]
*Structural features*				
Soma	Normal	Hypertrophic	Normal	
Dendrites	n.a.	Hypertrophic	Normal	
Spines	Increased	n.a.	Normal	
Axon	Varicosities	Varicosities	n.a.	
*Functional features*				
spont. firing	Decreased	Decreased	Normal frequency, increased irregularity	n.a.
Excitability	Decreased	Decreased	n.a.	n.a.
spont. EPSCs	n.a.	n.a.	n.a.	Decreased frequency
PF-EPSCs	Normal	Hyperfunctional	Normal	n.a.
PF-LTD	n.a.	n.a.	n.a. (but normal in the global KO)	Normal
CF-EPSCs	Normal	Hypofunctional	Normal	n.a.
GABAergic IPSCs	Normal	n.a.	n.a. (increased IPSC frequency in the global KO)	n.a.
